# The Relative Contribution of High-Gamma Linguistic Processing Stages of Word Production, and Motor Imagery of Articulation in Class Separability of Covert Speech Tasks in EEG Data

**DOI:** 10.1007/s10916-018-1137-9

**Published:** 2018-12-18

**Authors:** Amir Jahangiri, Francisco Sepulveda

**Affiliations:** BCI+NE Laboratory, School of Computer Science and Electronic Engineering, University of Essex, Colchester, UK

**Keywords:** Brain-computer interfaces, EEG, Linguistic processing stages, Motor imagery of articulation, Gabor transform, Davies-Bouldin index

## Abstract

Word production begins with high-Gamma automatic linguistic processing functions followed by speech motor planning and articulation. Phonetic properties are processed in both linguistic and motor stages of word production. Four phonetically dissimilar phonemic structures “BA”, “FO”, “LE”, and “RY” were chosen as covert speech tasks. Ten neurologically healthy volunteers with the age range of 21–33 participated in this experiment. Participants were asked to covertly speak a phonemic structure when they heard an auditory cue. EEG was recorded with 64 electrodes at 2048 samples/s. Initially, one-second trials were used, which contained linguistic and motor imagery activities. The four-class true positive rate was calculated. In the next stage, 312 ms trials were used to exclude covert articulation from analysis. By eliminating the covert articulation stage, the four-class grand average classification accuracy dropped from 96.4% to 94.5%. The most valuable features emerge after Auditory cue recognition (~100 ms post onset), and within the 70–128 Hz frequency range. The most significant identified brain regions were the Prefrontal Cortex (linked to stimulus driven executive control), Wernicke’s area (linked to Phonological code retrieval), the right IFG, and Broca’s area (linked to syllabification). Alpha and Beta band oscillations associated with motor imagery do not contain enough information to fully reflect the complexity of speech movements. Over 90% of the most class-dependent features were in the 30-128 Hz range, even during the covert articulation stage. As a result, compared to linguistic functions, the contribution of motor imagery of articulation in class separability of covert speech tasks from EEG data is negligible.

**Electronic supplementary material:**

The online version of this article (10.1007/s10916-018-1137-9) contains supplementary material, which is available to authorized users.

## Introduction

Speech is the most natural and intuitive form of human communication. Language and cognition are closely related processes. A BCI system designed to understand commands covertly spoken in the user’s mind, is highly desirable. Most neocortical territories in both hemispheres, as well as many subcortical brain regions are involved in language [1]. EEG signals can successfully identify 200–600 Hz cortical spikes [2–4] for medical diagnostic applications. In artefact-free conditions, EEG signals accurately measure induced/evoked high-Gamma brain activity, up to 150 Hz [5–8]. Based on the unique cognitive Neuroanatomy of each individual, the spatial, temporal, and spectral patterns of activity may vary from person to person [9].

Word production begins with semantic (conceptual preparation), lexical (Lemma retrieval), and phonetic (phonological code retrieval and syllabification) linguistic processes, followed by planning the movements of language muscles (phonetic encoding) for articulation [10, 11].

Linguistic phonetic processing is an automatic brain function, which elicits high-Gamma (70–160 Hz) oscillations [12, 13]. In each individual, Phonetic processing activity for a specific word does not change over time [14, 15] and is not affected by priming, cognitive activity, or task frequency [16, 17]. In contrast, semantic and lexical processing, is affected by task frequency, priming, and cognitive activity [18–20], which would also arbitrarily shift the temporal course of all following functions. These problems can be avoided by using a suitable experimental protocol.

**Fig. 1:**
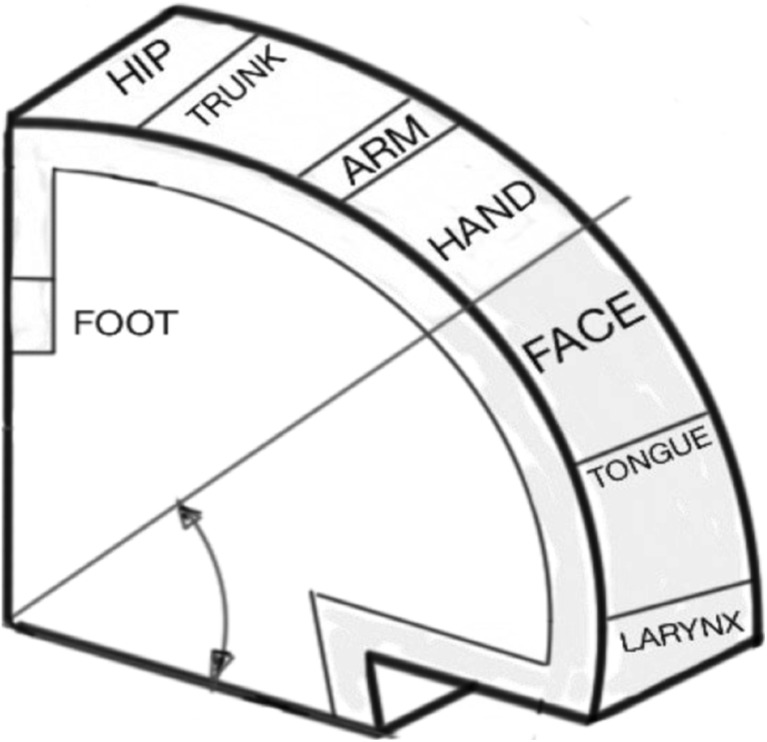
The functional division of primary motor cortex. A significant proportion, controls muscles responsible for speech

In covert speech, the manner of articulation in an individual (their ‘accent’) is consolidated over time. Covert articulation tasks activate the same language motor centres as their overt form [21, 22]. As a result, covert speech is produced with the same consistency as overt speech. However, in covert speech, the activity of the Primary Motor Cortex is greatly attenuated [23] and may be difficult to detect by EEG. Figure 1, illustrates the functional division of the primary motor cortex, also known as the “Homunculus”. Speech production is the most complex motor skill, which takes many years to learn and master. Almost one third of the Primary Motor Cortex is allocated to muscles producing speech, which reflects this complexity [24].

**Fig. 2:**
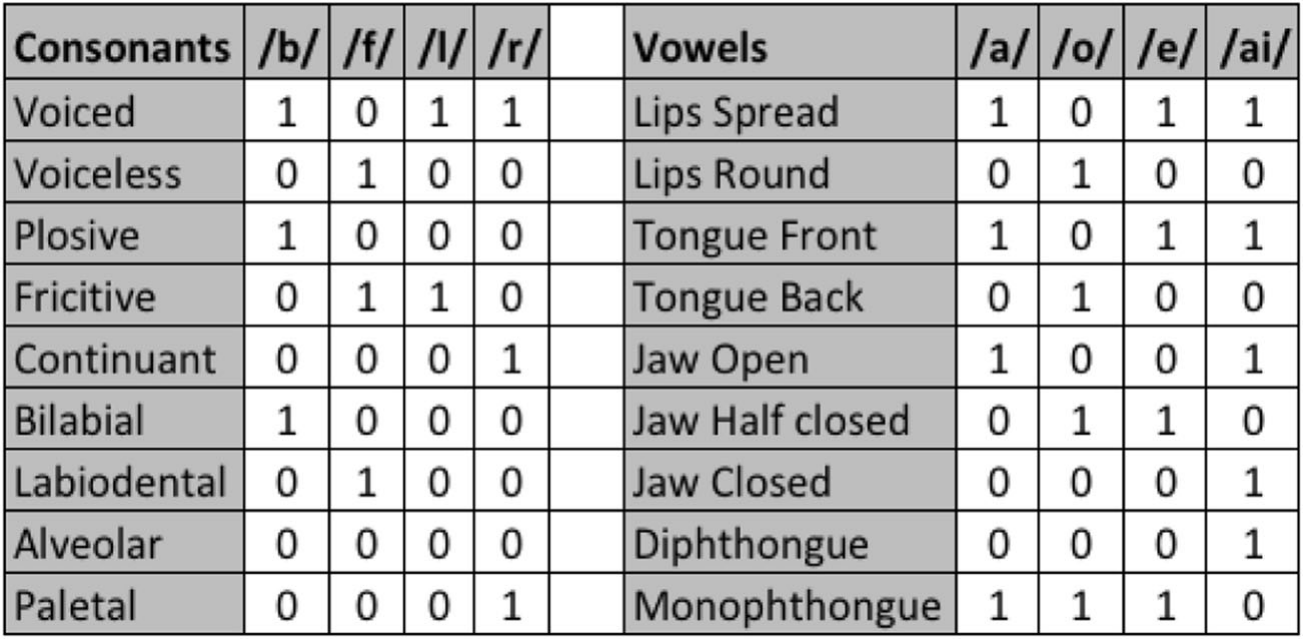
Properties of the consonants and vowels in the word classes, such as place of articulation and manner of articulation

Phonetically dissimilar covert speech tasks create distinctive neural activity associated with the phonological code retrieval and syllabification stages of linguistic processing [25] and involve different language muscle combinations during covert articulation. A linguistic BCI with four classes is sufficiently capable of controlling a smart device with a suitable user interface. In this study, the four directions (back, forward, left, and right) are shortened into Phonemic structures “BA”, “FO”, “LE”, and “RY” and used as covert speech tasks. These covert speech classes are cognitively appropriate directional commands, have little or no overlap with typical mind-wandering states, and provide an intuitive method of communication. For example, the user can move a cursor to the left by covertly speaking “LE”. In addition, these Phonemic structures are phonetically dissimilar. To demonstrate these phonetic differences in an accurate and quantitative manner, the properties of each consonant and vowel [26], such as place of articulation and manner of articulation, are presented in Fig. 2 [27]. For example, the consonant /b/ is voiced, plosive, and bilabial.

If the word class is known by the user before the trials, the conceptual preparation stage will be completed in advance. The Lemma selection stage, with multiple competing lemmas will have temporal inconsistencies. If trials are recorded in blocks, only one Lemma is activated and selected. In block recordings, the same auditory time cue, in the form of a “beep’ sound, can be used for task onset in all word classes, thus eliminating class-dependent auditory evoked responses from trials. By consolidated the semantic and lexical activities, conceptual preparation and lemma selection are complete before task onset. As a result, trials only contain automatic phonetic linguistic processing stages, and will not be affected by the temporal inconsistency of cognitive activity. Mental effort causes activation of scalp and neck muscles [28], which can mask high-Gamma cortical components. In this work, no mental effort is required from the user during trials. These conditions can be easily reproduced for the online application of this Linguistic BCI, with the same block recordings used for training.

After cue recognition (~100 ms post-onset), the following stages are [23]: Lemma activation (~100-175 ms post-onset), phonological code retrieval (~175-250 ms post-onset) and syllabification (~250-300 ms post-onset). Covert articulation (~500-800 ms post-onset) and the corresponding Motor imagery activity, are separated from the linguistic stages by a ~200 ms interval, during which covert articulation is designed by an internal perceptual process using the working memory and the somatosensory association cortex [9]. Initially one-second trials are used. By using shorter trials (0-312 ms post-onset), the covert articulation stage can be excluded from analysis to study its contribution to classification accuracy.

## Methods

**Fig. 3:**
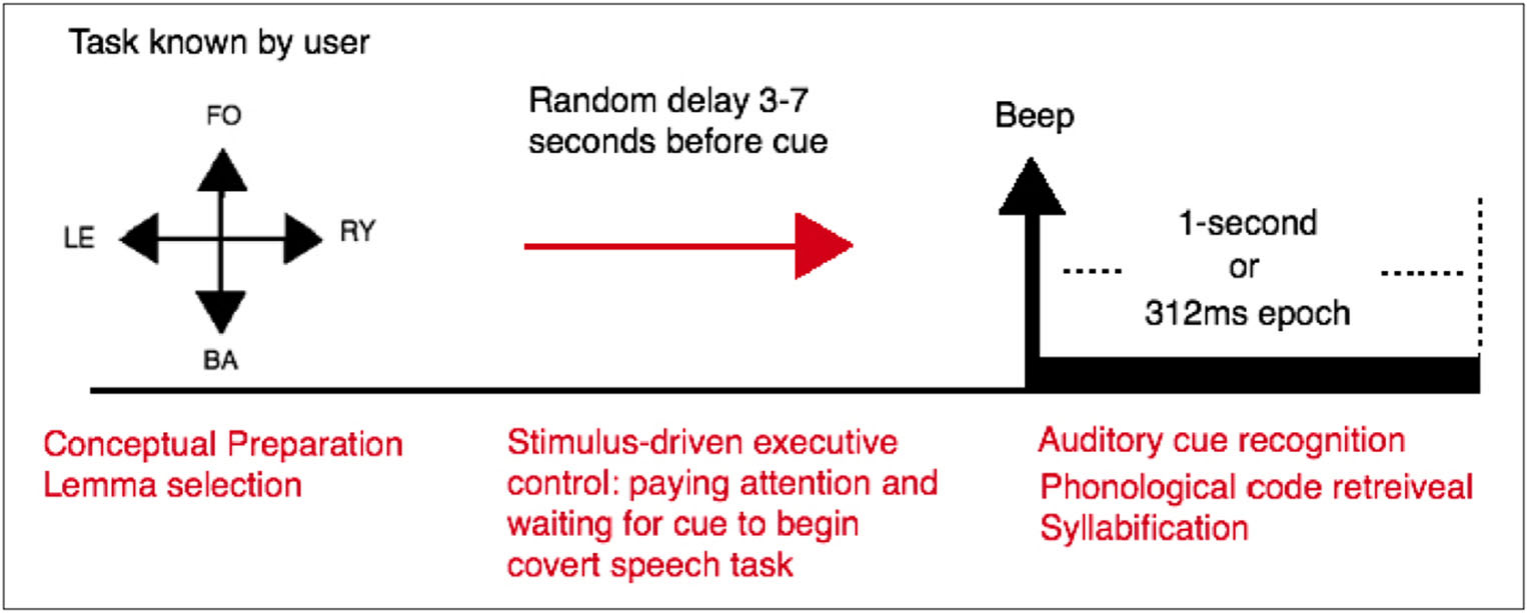
Imagination protocol. The user imagines speaking a word when an auditory cue in the form of a beep is presented. One second after each cue are used for the first experiment, and 312 ms for the second. A random rest period of 3–7 s occurs between trials. This sufficiently separated the tasks from one another. Also, the random duration prevents the user from anticipating the task onset based on rhythm. As a result, the next stages of linguistic functions begin exactly after cue recognition and the system is perfectly synchronised

This study was conducted with 10 neurologically healthy volunteers in the age group of 21–33. All volunteers signed a consent form based on the recommendations of the Ethical Committee of the University of Essex. Participants were seated in a comfortable armchair. The experiment consists of 4 recording runs for a participant, each containing 30 trials of only one class. For all classes, an identical “beep” sound was used as the auditory cue. The user was informed of the task before each run and asked to covertly speak when they heard the timing cue. As a result, Conceptual Preparation, and Lemma selection are completed before onset. A random rest period between 3 and 7 s was placed between trials to prevent the user from anticipating onset time based on rhythm. This ensures the remaining linguistic activities (Phonological Code Retrieval, Syllabification, Covert Articulation) begin exactly after auditory cue recognition, and the system is perfectly synchronised. Recent studies on the time windows of the processing stages of language production provide evidence of latent activities of over 2000 ms [9]. The 3–7 s idle period sufficiently separates the trials. Figure 3 shows the imagination protocol of the experiment.

The EEG signals were recorded using a 64 channel Biosemi ActiveTwo™ system [29]. One computer generated the graphical user interface and sent trigger signals to the ActiveTwo device at the instant a time cue was presented to the user. The triggers were sent via the parallel port and were visible in the recorded data. A second computer saved the EEG recordings and was connected to the ActiveTwo’s A/D box via USB. Electrode placement was done per the international ABC system, which for 64 channels corresponds to the 10/10 system. The ActiveTwo has a pre-amplifier stage on the electrode and can correct for high impedances. However, the offset voltage between the A/D box and the body was kept between 25 mV and 50 mV as recommended by the manufacturer. The data were recorded at a sampling rate of 2048 samples/s, with guaranteed data frequency content of 0-409 Hz according to BioSemi.

The pre-processing was done with the use of EEGLAB [30], an open source MATLAB™ toolbox. Studies conducted with the use of intra-cranial implants confirm high gamma band activity during covert speech tasks [20, 31, 32]. One of the main reasons that numerous studies have failed in achieving high classification accuracy, is that covert speech tasks are treated as motor imagery, and information above the beta band is often ignored or even filtered out [33]. A suitable frequency range (0-128 Hz) for analysing Linguistic activity is achieved by down-sampling the data to 256 Hz. This frequency range is within the operating capability of the ActiveTwo system. The data is then referenced using surface Laplacian. To remove 50 Hz noise from UK power lines, a FIR notch filter, with rejection band of (49.2–50.8 Hz) was applied. Using the Automatic Artifact Removal (AAR) toolbox in EEGLAB [34], EOG and EMG artifacts were reduced, with SOBI [35] and CCA algorithms [36] respectively. These methods outperform ICA, which is ineffective beyond 70 Hz [37, 38]. Unfortunately, no algorithm can completely eliminate EMG, which elicits 20-200 Hz oscillations in EEG [28, 39]. The most effective solution is to reduce the possibility of recording EMG by controlling the experiment protocol and the environment. The final stage of pre-processing is extracting epochs from the continuous EEG recordings. Each epoch begins when beep sound is generated and ends exactly one second (or 312 ms for shortened trials) later.

**Fig. 4:**
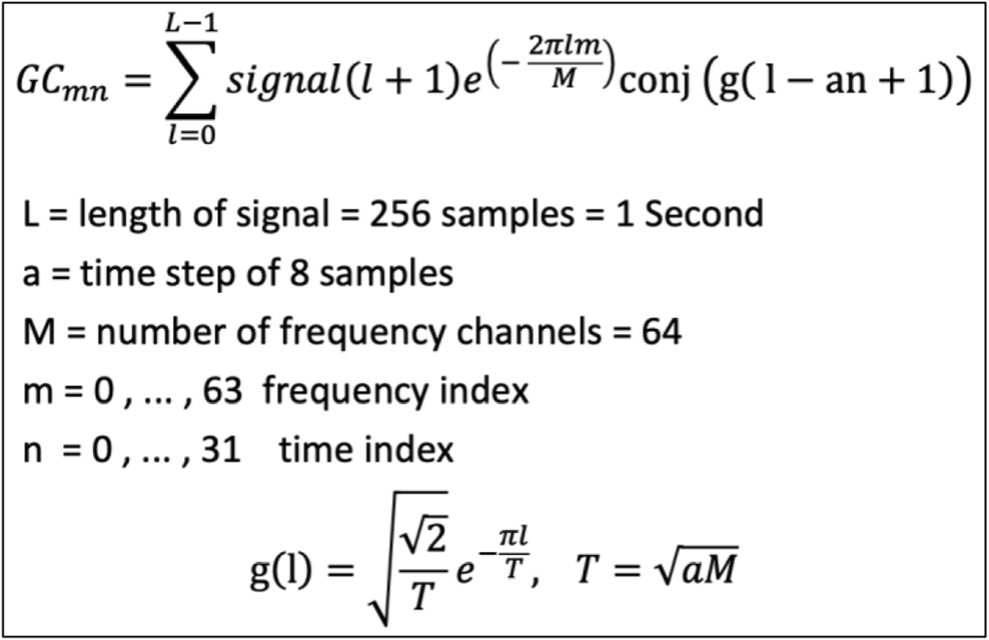
Definition of Gabor coefficients by implementation of the direct discrete Gabor transform and a Gaussian window function

This work is a novelty search with an exploratory approach. The experimental data were processed offline and the main objective was to initially create a detailed feature space, in such a way that little or no relevant information is lost or excluded. Features must contain information on time and frequency and should maintain their link to EEG channel for possible topographical analysis. The discrete Gabor Transform [40, 41] (presented in Fig. 4) was thus used as it satisfies all these requirements. Although the Gabor transform is computationally taxing, it has been successfully applied to find hidden information in EEG data with muscle artefact noise contamination to predict onset of seizures [42, 43] and to identify the location of seizure sources [44]. The Gabor transform has also been used for feature generation to classify motor imagery tasks that are very similar, such as different movements of the same hand [45, 46]. In the present study, a time step of 0.03125 s (32 steps per second) and frequency band of 2 Hz (64 frequency bands) were chosen to provide the best tradeoff between classification performance and computational cost.

A 1-s epoch from a single EEG channel (256 samples) is converted into a 64 × 32 feature matrix. For the 312 ms trials (80 samples), one epoch from one channel is converted into a 64 × 10 feature matrix.

**Fig. 5:**
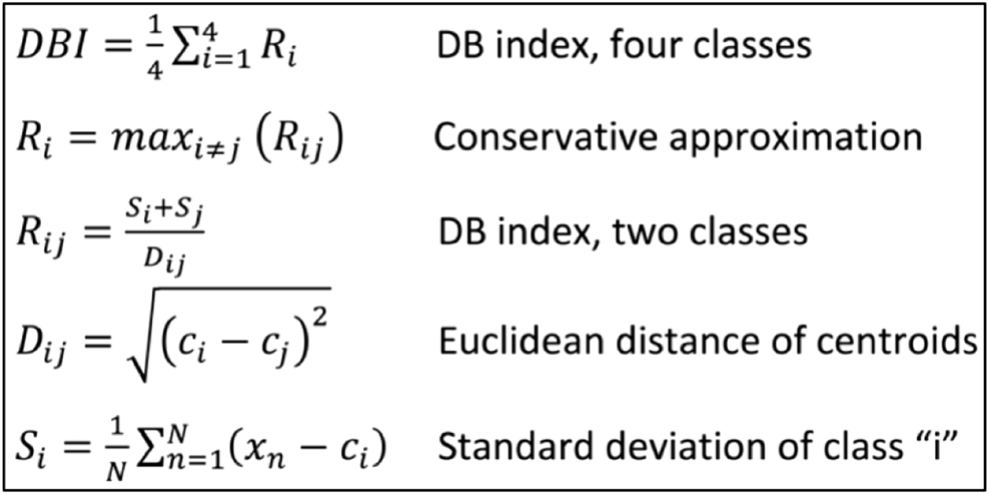
Definition of the Davies-Bouldin index for 4 one-dimensional clusters. Most valuable features have the smallest DBI

To maximise classification accuracy, it is necessary to identify the most distinctive features between the four covert speech classes and use these features to train the classification object. Dimensionality reduction and feature selection with clustering algorithms is proven to be extremely effective [47–49]. The Davies-Bouldin index [50] is a function of within-cluster scatter to between-cluster separation [51, 52], and can be used to determine most useful features to distinguish the four word classes. DBI matrices for all the six word-pairs (e.g., BA vs. FO) are calculated, and used to assign a conservative value to each feature in the “one-vs-all” DBI. Features with the lowest DBI index are considered the most valuable for class separation. Figure 5 shows the definition of the Davies-Bouldin index with four one-dimensional clusters.

**Fig. 6:**
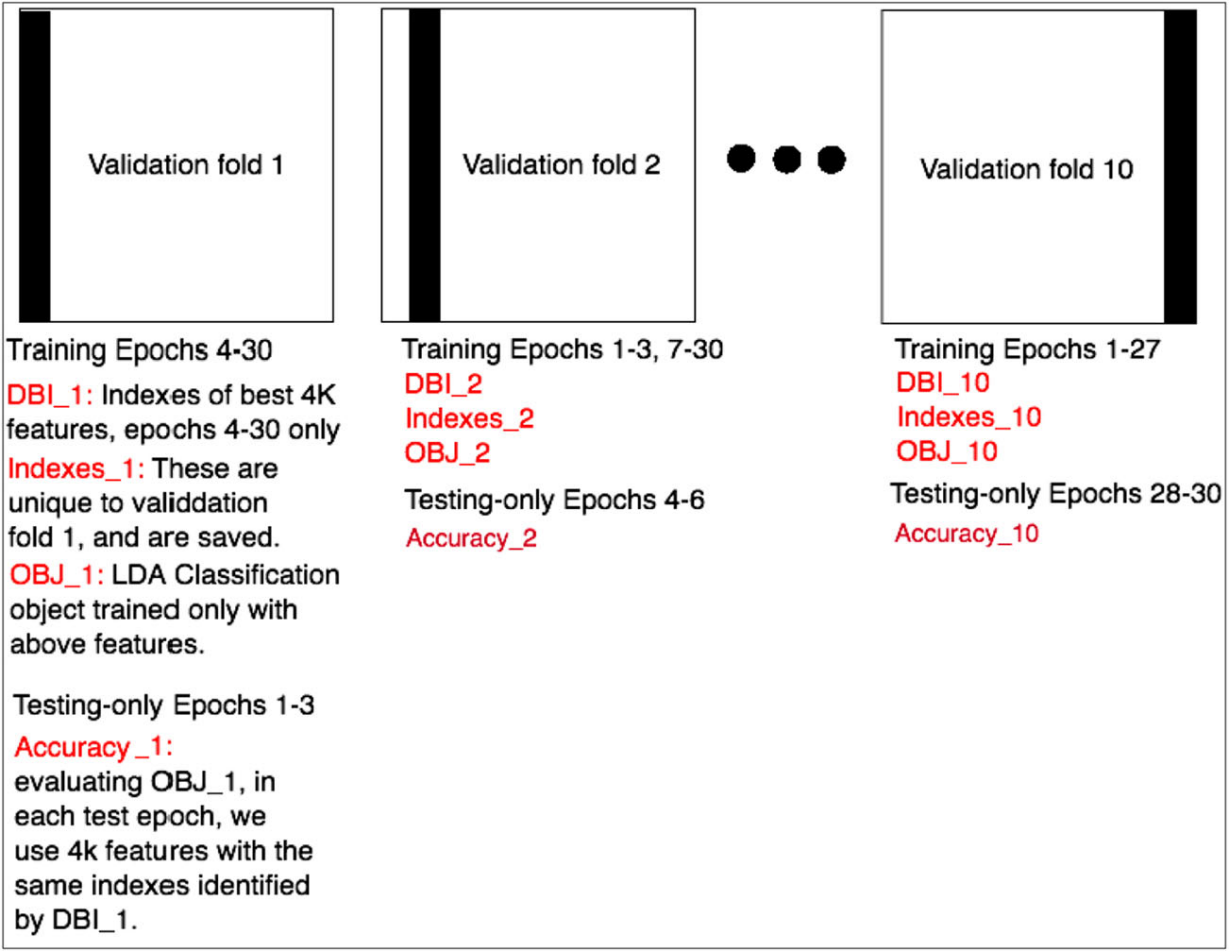
The process of cross validation, feature selection, training, and testing used in this work is presented here. The grand average true positive rate is the mean and standard deviation of “Accuracy_1” through “Accuracy_10”

The mean and standard deviation of a 10-fold cross validation process [53] were used to estimate the true positive rate. For each validation fold, 27 trials were used for training, and 3 remaining trials were set aside for testing only. Testing trials change from one validation fold to the next, and over 10 folds, all 30 trials are used in testing. The process of cross validation, feature selection, training, and testing used in this work is presented in Fig. 6.

Only the feature generation stage, using the discrete Gabor transform, is applied to the entire dataset. All other calculations are unique and fold-dependent. In this study, for the 1-s trials each DBI matrix has a dimension of 4096 × 32 (64 frequency-bands, 64 channels, 32 time-steps). Based on the DBI, features are ranked and sorted in order of importance. The indexes of the most valuable 4000 features are saved, and these features used for training the classification object. This filtering approach for feature selection reduces the dimensionality of the feature space by 97%, with acceptable computational cost. The 312 ms trials use the same analysis pipeline as 1-s trials. For 64 channels, the dimension of the DBI matrix for 312 ms trials is 4096 × 10 (64 frequency bands, 64 channels, 10 time-steps).

Pseudo-Linear discriminant analysis was applied for classification, as it consistently out-performed all other supervised machine learning methods, for EEG recorded covert speech data [54]. Compared to the training process, the computational cost of testing is negligible.

## Results

**Fig. 7:**
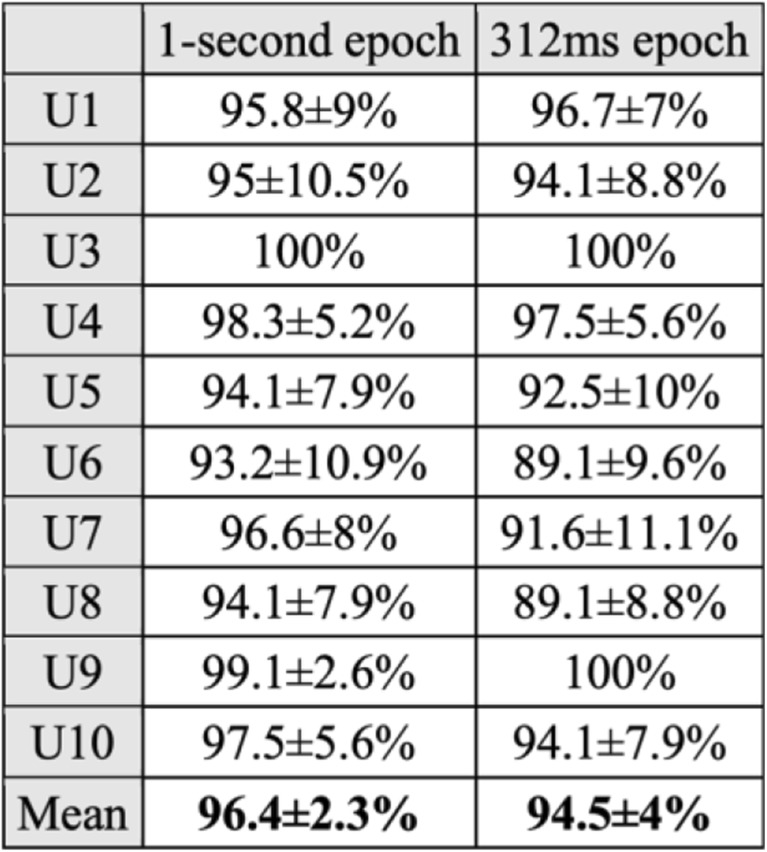
The true positive rates of one word vs. all, estimated by a ten-fold cross validation method. Eliminating the covert articulation stage from analysis has less than 2% effect on grand average classification accuracy. Considering the Wilcoxon *p* value of 0.9269, compared to the high-Gamma linguistic processing stages, the contribution of motor imagery of articulation in class separation of covert speech tasks from EEG data is negligible

The true positive rates of one word vs. all, are generated by a standard ten-fold cross validation method. Figure 7 presents these values for 1-s epochs, and for 312 ms epochs. By eliminating the covert articulation stage from trials, the relative contribution of Motor Imagery of speech and linguistic processing stages, in classification accuracy can be determined.

The Wilcoxon rank-sum test on both columns returns a p value of 0.9269. By using 312 ms trials instead of 1-s trials to exclude covert articulation, the computational cost is reduced to one third, with less than 2% penalty in classification accuracy. During covert speech, the language motor regions are suppressed, but not completely deactivated [23]. As a result, during the covert articulation stage, there will be minute involuntary muscle movements related to each phonemic structure, which will create class-related, high-Gamma Myoelectric artefacts. The 312 ms trials are complete before the covert articulation stage begins (~500 ms post onset) and are guaranteed to be free from class-related EMG. Possible involuntary early muscle ticks (i.e. lip movements ~160 ms after cue) can cause significant EMG contamination. The CCA algorithm used here, only removes such artefacts from the first 400 ms of data (312 ms trials included) [55].

**Fig. 8:**
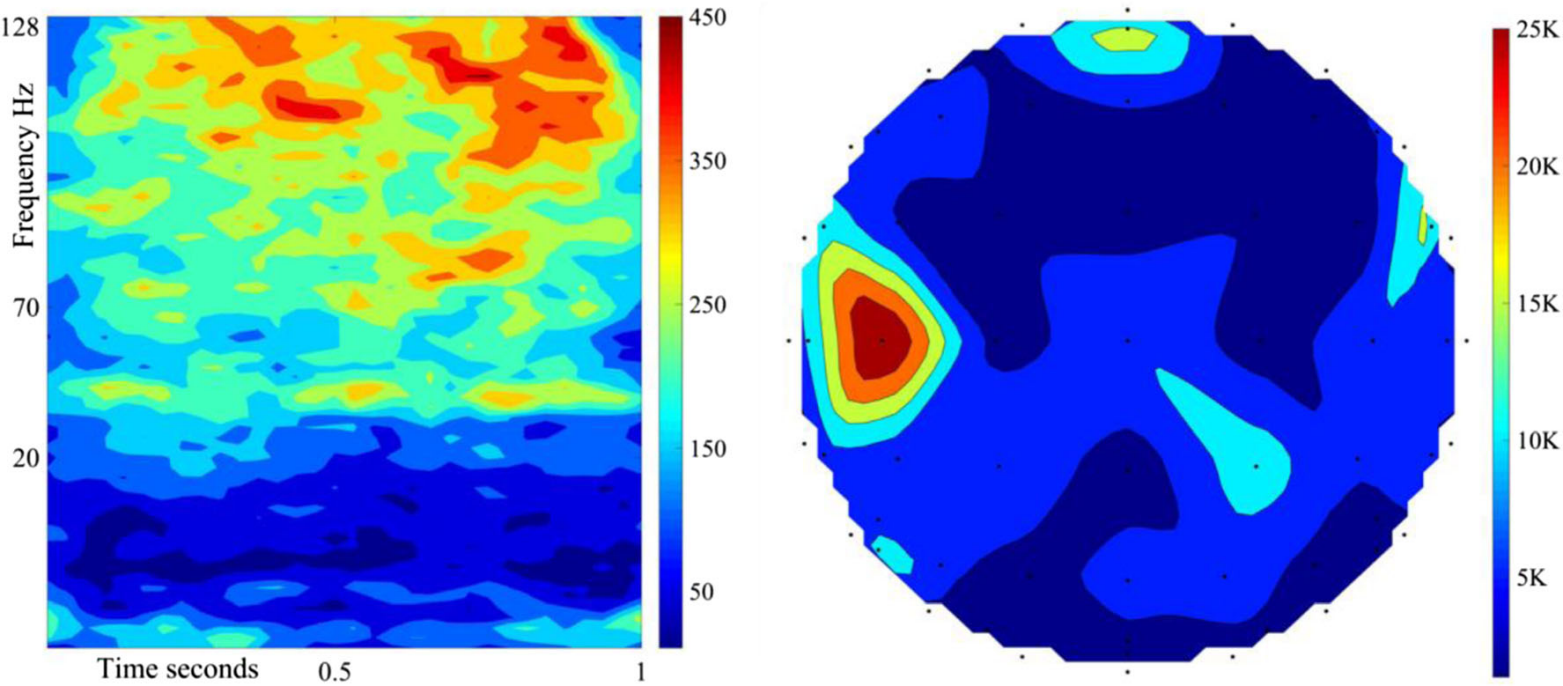
The cumulative colour-coded joint time-frequency representation of 4e5 features from 10 users, 1-s trials (Left). The associated topographical plot (Right). The top of the plot is the front of the head. The greatest concentration is within 70–128 Hz

From 10 users, 10 validation folds/user, and 4000 features/fold, 4e5 best features are identified from the experiment with 1-s trials, and 4e5 from the shortened 312 ms trials. Each Gabor feature is linked to a frequency band, time step, and EEG electrode. The 4e5 features identified in the 1-s trials are cumulatively placed in the 64 × 32 feature space to create a colour coded time-frequency representation of the most class-dependent Neural activity, and to identify the electrodes recording this activity for a topographical map of the brain [56, 57]. These plots are illustrated in Fig. 8. The features are highly concentrated in the 70-128 Hz band, even during the covert articulation stage.

**Fig. 9:**
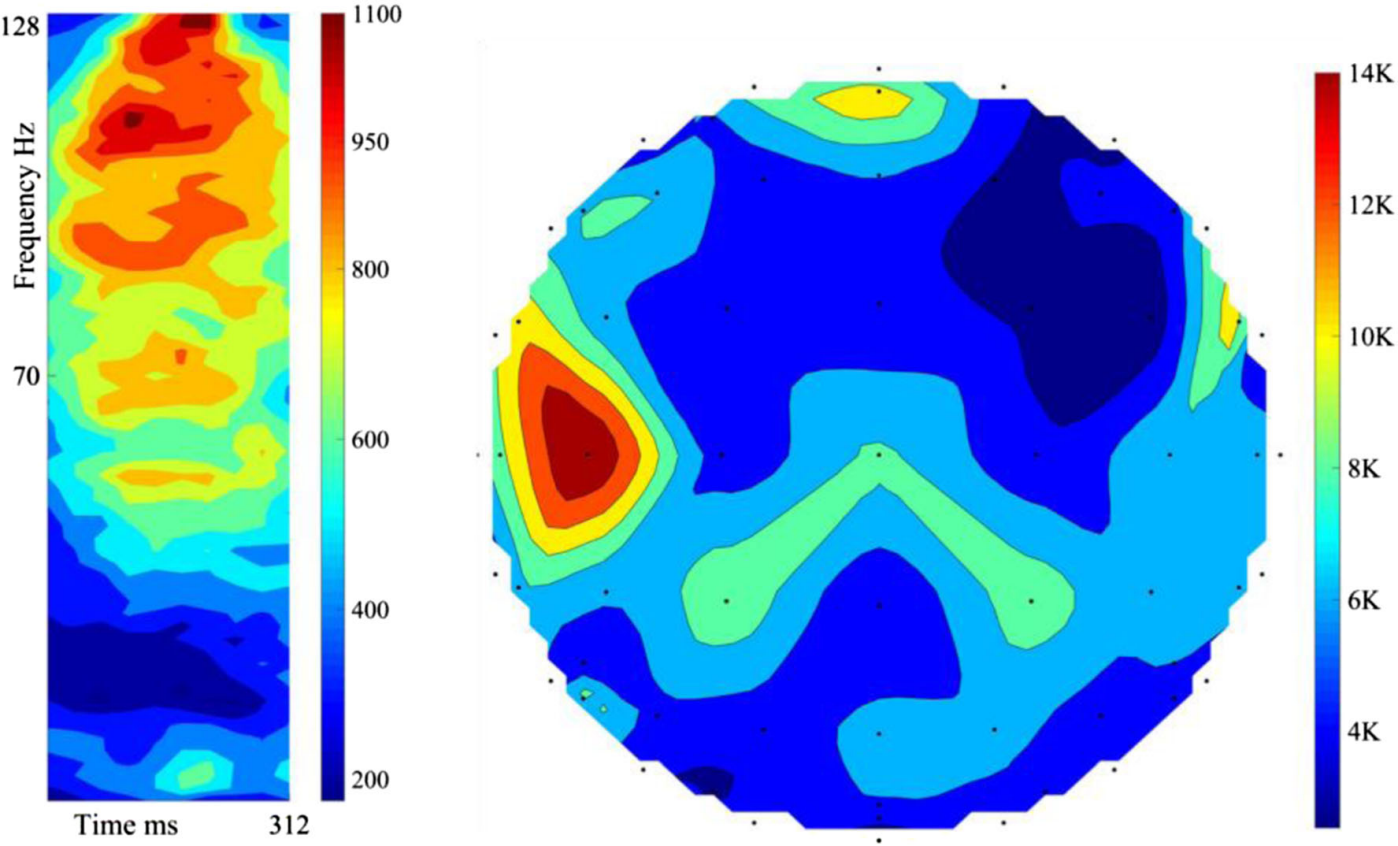
The cumulative colour-coded joint time-frequency representation of 4e5 features, 312 ms trials (Left). The associated topographical plot (Right). Most important regions: Prefrontal Cortex, left STG (Wernicke’s area), right, and left IFG (Broca’s area)

The 4e5 features identified in the 312 ms trials are cumulatively placed in the 64 × 10 feature space to create a colour coded time-frequency representation and used to create a topographic brain map (Fig. 9). The most significant regions are the Prefrontal Cortex [58] (stimulus driven executive control), the left Superior Temporal Gyrus [9] (Wernicke’s area, phonological code retrieval), the right, and left Inferior Frontal Gyrus [9] (Broca’s area, syllabification). The same regions are prominent in both Figs. 8 and 9.

## Discussion

In a recent publication by these authors [59] an identical experimental protocol and analysis pipeline to this work were used to record mixed randomised trials in a single run using an Enobio dry electrode system with 20 channels. To achieve a manageable recording duration (6–7 min), only 20 trials were recorded per class, and the idle period between trials was reduced to 1–3 s. A grand average classification accuracy of 85% was achieved. Despite using fewer channels, inferior electrodes, and fewer trials compared to the current work, the system performed extremely well for mixed randomised recordings.

**Fig. 10:**
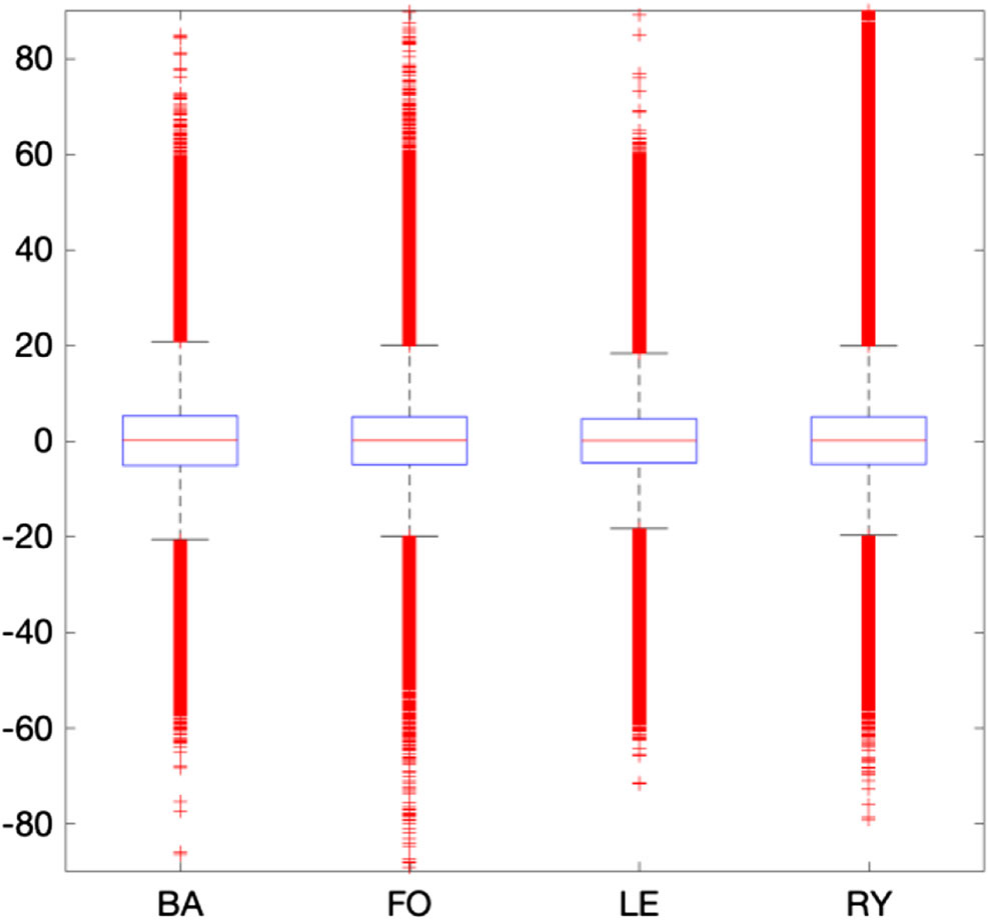
The distribution properties of raw EEG recordings in each block for user 1. In all blocks, the mean is 0, std. is 10, the 25% and 75% quartiles are −20 and 20 respectively, and range is near 180. They all have Gaussian distribution. With classification accuracy of 96.7%, no signs of signal drifting exist, suggesting that recording in blocks has little, if any effect on classification accuracy for this data

Recording 120 trials in a single run using the experimental protocol presented in this work, requires 25–30 min. Maintaining constant focus for such a long duration is exhausting for the user. To reduce user fatigue, trials were recorded in four blocks, each 7–8 min in duration. For each user, the distribution properties (mean, std., rang, etc.) of the raw EEG recordings are virtually identical in all four blocks. Figure 10 presents the distribution properties of the recorded blocks from user 1. The classification accuracy for user 1 is 96.7%. The raw recordings for all 4 blocks closely match each other’s distribution properties. This indicates there are no drifts in the recorded signals (i.e. change of an electrode’s impedance) causing positive bias in classification accuracy.

**Fig. 11:**
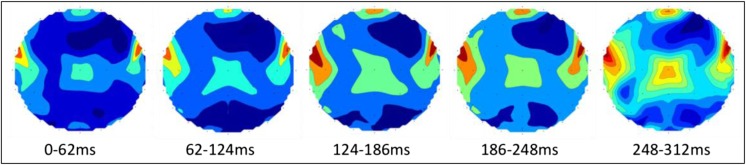
Topographical maps of brain regions generating the most distinctive features within the indicated 62 ms interval. The plot for the 248-312 ms interval indicates the early stages of perceptual planning, before activation of the SMA (~500 ms) and covert articulation

The topographical map in Fig. 9 shows the overall activity up to 312 ms post task onset. To demonstrate the sequence of activations, topographical plots with 62 ms intervals are created (Fig. 11). Each plot only contains features from the indicated time range. The sequence of activation is as follows [9]:

- [0-62 ms] Left, and right Auditory Cortex: response to auditory cue.
- [62-124 ms] Prefrontal Cortex [58]: Stimulus-driven executive control, initiating covert speech with auditory cue recognition (100 ms). Left Middle Temporal Gyrus: Lemma activation (100-124 ms).
- [124-186 ms] left Superior Temporal Gyrus: Phonological code retrieval.
- [186-248 ms] Left and right Inferior Frontal Gyrus: syllabification.
- [248-312 ms] Left inferior, and Superior Parietal Cortex [58]: Goal-driven executive control, by suppressing the Primary Motor Cortex, and activating an internal perceptual planning process [60–63].

The syllabification stage is completed sooner than estimated, and the 312 ms trials contain the very early stages of perceptual planning. However, the covert articulation stage, which occurs after the activation of the Supplementary Motor Area [9, 64], is excluded from shortened trials as intended. In the 312 ms trials, the spatial, temporal, and spectral properties of the 4e5 most valuable features identified from 10 participants (Figs. 9 and 11), correspond to the automatic linguistic processing stages of word production prior to articulation, and are supported by a substantial body of evidence [9, 10, 12–15, 20–22, 25, 31, 32, 60]. This, in addition to eliminating the possibility of drifts in the raw EEG recordings, confirm the validity of our findings.

## Conclusions

By excluding motor imagery, grand average classification accuracy dropped from 96.4% to 94.5%. Compared to the high-Gamma linguistic processing stages of word production, the contribution of motor imagery of articulation in class separability of covert speech tasks is negligible. However, by using 312 ms trials instead of 1-s trials, the computational cost is significantly reduced. The 312 ms trials used in this work, only contain phonetic linguistic processing activity. Phonetic linguistic processing prior to articulation, elicits a unique and word-specific pattern of high-Gamma activity [12, 65], which does not change over time [14, 15] and is not affected by frequency [16] or priming [17]. Phonetic codes are set up and consolidated with the acquisition of language during childhood, and remain unchanged throughout a person’s life [17]. Phonetic codes are stored in the long term memory, and are processed automatically by the brain requiring no conscious effort from the user during trials, with immunity from any influence or modification [16, 17, 65, 66]. The experimental protocol and analysis pipeline for 312 ms trials presented in this work can be used as a framework to create an online EEG-based 4-class linguistic BCI in future studies. The raw EEG recordings for all ten participants in this work have been published on “Mendeley Data” (10.17632/5c2z92vw3g.2) for the benefit of our readers.

## Electronic supplementary material

ESM 1 (PDF 366 kb)
