# Supplementary material for: The Relative Contribution of High-Gamma Linguistic Processing Stages of Word Production, and Motor Imagery of Articulation in Class Separability of Covert Speech Tasks in EEG Data
Source: J Med Syst. 2018 Dec 18;43(2):20. doi: 10.1007/s10916-018-1137-9 (PMC6299054; doi:10.1007/s10916-018-1137-9)
Supplement: Supplementary file 1 — (PDF 366 kb) [file 10916_2018_1137_MOESM1_ESM.pdf]

Appendix A

Covert Speech vs. Motor Imagery: a comparative study of class separability in identical environments

Amir Jahangiri (ajahan@essex.ac.uk), Juan M. Chau (jc18230@essex.ac.uk), David R. Achanccaray (dachanccaray@pucp.pe), Francisco Sepulveda (fsepulv@essex.ac.uk). BCI research Laboratory, School of Computer Science and Electronic Engineering, University of Essex, UK.

***Abstract*— In this study a single experimental protocol and analysis pipeline is used: once for MI tasks, and once for covert speech tasks. The goal of this study is not to maximizing classification accuracy; rather the main objective is to provide an identical environment for both paradigms, while identifying the most important activities related to the most class dependent features. Four volunteers participated in this experiment. With four classes, the average classification accuracy for covert speech tasks is 82.5%, and for motor imagery is 77.2%. The average performance is significantly higher than chance level for both paradigms, suggesting that the results are meaningful, despite being imperfect. For motor imagery tasks the most important activities are the execution of imagined movements, and goal driven executive control for suppression of overt movements, which also occur for covert speech tasks. However, the most important activity for covert speech tasks is the linguistic processing stages of word production prior to articulation, which does not occur in motor imagery. These high-Gamma linguistic processes are extremely class dependent, which contribute to the higher performance of covert speech tasks, compared to motor imagery in an otherwise identical environment.**

I. Introduction

Motor imagery is a well-established paradigm in BCIs. The low-frequency oscillations (< 35 Hz) elicited by "MI" activity, have been detectable by EEG for many decades. MI does not occur independently and is the end-result of many cognitive functions. For example, anticipating an onset cue and initiating "imagined" movement after cue recognition requires stimulus-driven executive control, with high-Gamma activity in regions such as the pre-frontal cortex [1, 2]. To take advantage of such class dependent cognitive activity [3, 4], the entire data bandwidth of the EEG system must be utilized [5] (and not only Alpha and Beta bands). Covert word production begins with high-Gamma (>70 Hz) linguistic processing stages [6-8], followed by motor imagery of articulation [9, 10]. Language is exceedingly more complex than movement [11] and requires analysis with much higher resolution than traditional MI band power [12]. However, covert speech is more intuitive and natural for BCI communication compared to MI. In this study, a single experimental protocol and analysis pipeline is used: once for MI tasks, and once for covert speech tasks. The performance of the system for each paradigm is calculated and the results are discussed.

II. Methods

A. Experiment Protocol

In this study, each recording run contains four classes, which are shown in the user interface by four arrows: up, down, left, and right. Within a recording run, 10 examples of each task are presented in a random order (each run has 40 trials) to avoid user fatigue. During recording, a new task is determined by an arrow appearing on the screen for 3 seconds. After the arrow disappears, there is a 3 second standby state. Task onset is presented as a beep sound for all classes. A second beep indicates a rest period before the next trial. The experimental protocol is presented in figure 1.

Each user completes two recording runs, which are identical in every way with the exception of type of mental task (MI, covert speech). For MI tasks, the four arrows represent left hand movement (left arrow), right hand (right arrow), left foot (down arrow), and right foot (up arrow). In covert speech tasks, the user imagines speaking the phonemic structures: BA (back/down arrow), FO (forward/up arrow), LE (left arrow), and RY (right arrow), which are phonetically very dissimilar tasks [13].

B. Data Acquisition

Four neurologically healthy volunteers participated in this experiment. The EEG signals were recorded using an Enobio dry electrode system with 20 channels and 10/10 configuration [14]. Data was recorded at a sampling rate of 500 Hz and saved in "gdf" format. Compared to wet electrode systems, setting up the Enobio is extremely easy. However, the quality of recorded signals may restrict the number of classes it can use simultaneously. This study provides an evaluation of the system's capability.

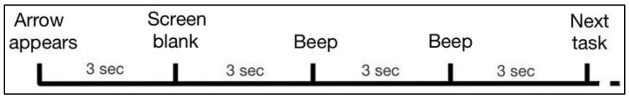

Figure 1. The experiment protocol for recording four randomly presented trials. Each class corresponds to a directional arrow. After task presentation, a beep sound is used for all classes as task onset. A second beep indicates a rest period before the next task.

C. Pre-Processing

Recorded data was pre-processed using EEGLAB [15]. Data was down-sampled to 256Hz and re-referenced using common average. Line noise was removed with an FIR notch filter (49.5,50.5Hz). The AAR toolkit [16] was used for artefact rejection. EOG and EMG artifacts were reduced, with SOBI [17] and CCA algorithms [18] respectively. These methods outperform ICA, which is ineffective beyond 70 Hz [19, 20]. One-second epochs were extracted from the pre-processed data and saved as a numeric matrix for further analysis.

D. Feature Extraction

The discrete Gabor transform [21, 22] was used to generate features. The original data can be reconstructed from the features with no information loss. Each Gabor feature contains information on both time and frequency. In this study, a time step of 0.015625 seconds (4 time samples) and a frequency band of 2Hz is used. A one-second epoch from one channel (256 time samples) is converted into a 64x64 feature matrix. Figure 2 presents the definition of the discrete Gabor transform. This method of feature selection makes it possible to identify the type of neural activity from the indexes of the features used in classification.

E. Feature Selection and classification

Classification true positive rate is estimated by a 5-fold cross validation process [23]. In each fold, 8 trials are used for feature selection and training the classification object, and 2 trials are set aside for testing. The most valuable features for distinguishing four classes are discovered by the Davies-Bouldin index [24]. Initially, all pair-wise DBI matrices are calculated (6 binary combinations with 4 classes). The four-class DBI is a conservative approximation based on the two-class DBIs, which is defined in figure 3. In this experiment, 91% of the total computational cost is spend on generating the DBI matrix. However, the dimensionality of the feature space is significantly reduced. In this study, the 3K most valuable features (from a total of 81920) are identified and used to train the LDA classifier. Features in the test data with the same indexes are used to test the performance of the classifier.

$$GC_{mn} = \sum_{l=0}^{L-1} signal\ (l+1)\ e^{(-2\pi\, lm/M)} conj\ (g(l-an+1))$$

L = length of signal = 256 samples = 1 second

a = time step = 4 samples

M = number of frequency channels = 64

m = 0 , ... , 63 frequency index

n = 0 , ... , 63 time index

$$g\ (l) = \sqrt{\frac{\sqrt{2}}{T}} * e^{-\pi\ (l/T)} \quad \text{Gaussian window ,} \quad T = \sqrt{a*M}$$

Figure 2. Definition of Gabor coefficients by implementation of the direct discrete Gabor transform and a Gaussian window function.

$DBI = \frac{1}{4}\sum_{i=1}^{4} R_i$ DB index, four classes

$R_i = max_{i\neq j}\ (R_{ij})$ Conservative approximation

$R_{ij} = \frac{S_i+S_j}{D_{ij}}$ DB index, two classes

$D_{ij} = \sqrt{(c_i - c_j)^2}$ Euclidean distance of centroids

$S_i = \frac{1}{N}\sum_{n=1}^{N}(x_n - c_i)$ Standard deviation of class "i"

Figure 3. Definition of the Davies-Bouldin index for 4 classes. The most valuable features have the smallest DBI.

III. Results

A. Classification Accuracy

The true positive rates for one class vs. all, are estimated as the mean and standard deviation of the five-fold cross validation process. Table 1 contains these results for the four participants and both types of cognitive task. The reader should kindly consider that the objective of this study is not to maximize classification accuracy. The experimental protocol and analysis pipeline provided identical environments for both paradigms, while identifying the most important activities related to the selected features. With four classes, the classification accuracy is significantly higher than chance level for both paradigms, suggesting that the results are meaningful, despite being imperfect.

B. Time-frequency distribution of best features

The 60K features identified in the motor imagery experiments, are shown in a cumulative joint time-frequency plot of the feature space and presented in figure 4. As expected, valuable class dependent activity is not limited to the Alpha and Beta bands. In addition, the nominal bandwidth of (1-125) Hz given by Enobio is confirmed, as valuable features are identified in the entire frequency range.

Table 1. True positive rates of one class vs. all. These are estimated using a five-fold cross validation process. With four classes, the average performance is significantly higher than chance level for both paradigms, suggesting that the results are meaningful, despite being imperfect.

	Covert Speech	*Motor Imagery*
User 1	85 ± 33.3	80.1 ± 32.7
User 2	80.5 ± 30.8	68.5 ± 28.1
User 3	87.3 ± 21.2	83.4 ± 33.4
User 4	78 ± 18.9	78 ± 30.9
Average	82.5 ± 24.1	77.2 ± 31.2

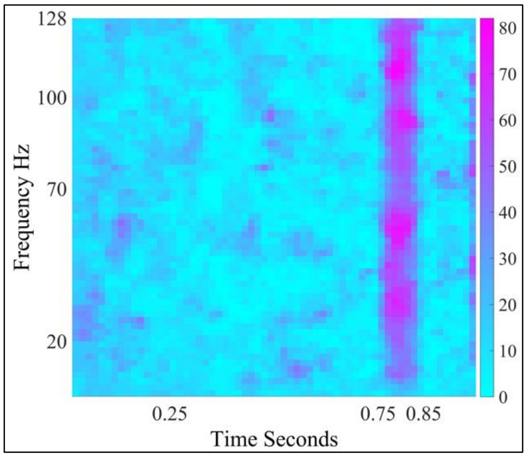

Figure 4. The cumulative joint time-frequency plot of the feature space containing the most valuable 60K features identified in the motor imagery experiments (4 users, five validation folds, and 3K features per fold). The (0.73,0.875) second band contains 15.1% of features.

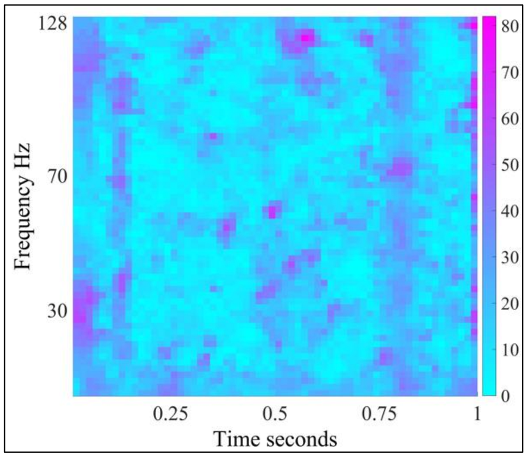

Figure 5. The cumulative joint time-frequency plot of the feature space containing the most valuable 60K features identified in the covert speech experiments (4 users, five validation folds, and 3K features per fold). 48.8% of these features are above 70 Hz. The (0.73,0.875) second band is not as prominent as the MI paradigm from figure 4.

For motor imagery tasks in this experiment 15.1% of all the most valuable features are significantly concentrated within the (0.73-0.875) second range. This time period corresponds with performing imagined movements and the suppression of the Primary Motor Cortex (stopping actual movements) via "goal driven executive control". Such executive control involves high-frequency cognitive activity in brain regions such as the Superior Parietal Cortex and the Pre-Frontal Cortex [1, 2]. 23.2% of all the most valuable features are within the Alpha and Beta bands (MI). The other 76.8% of the features are in the Gamma, and high-Gamma bands (cognitive functions). This suggests that in motor imagery tasks, cognitive functions generate a significantly greater amount of class dependent activity compared to the execution movement.

Figure 5 presents the cumulative joint time-frequency plot of the feature space containing the most valuable 60K features identified in the covert speech experiments. 48.8% of these features are above 70 Hz, which correspond with the linguistic processing functions [8]. These linguistic functions, which are entirely class dependent, do not exist in motor imagery. This provides a possible explanation for the higher classification accuracy of covert speech tasks (82.5%) compared to motor imagery tasks (77.2%) in an identical environment, considering there is a direct positive correlation (with R=0.8822 and P=0) between their performances.

Considering that tasks are identified before trials begin, the cognitively demanding linguistic functions (conceptual preparation, Lemma selection) are completed before onset. The linguistic functions occurring within trials (phonological code retrieval, syllabification) are performed automatically by the brain [9] and require no user effort. All other cognitive functions within trials (executive control, imagined movement) are also present in MI tasks. As a result, the cognitive effort of using covert speech tasks and MI tasks are virtually identical in this study.

IV. Discussion

The linguistic processing stages of word production prior to articulation, which are entirely class-dependent, consist of conceptual preparation, Lemma selection, phonological code retrieval, and syllabification [25]. By incorporating difference in meaning, and difference in phonetic structure, for selecting selected covert speech tasks, class separability can be significantly enhanced.

In this experiment, linguistic class separability is maximized by selecting phonetically dissimilar covert speech classes [13]. This explains the superior performance of covert speech tasks compared to MI tasks in the otherwise identical environment designed in this study.

Linguistic studies using intra-cranial implants have demonstrated that these linguistic processing stages have high-Gamma signatures in the (70-170Hz) range [10, 26-29]. As bandwidth of EEG systems increases and EMG removal algorithms become more reliable, covert speech BCIs will become much more capable. Although other BCI systems (such as MI) will also improve, language, which is the most intuitive and natural form of human communication, would logically be the preferred paradigm of choice for a BCI.

V. Acknowledgment

This work was supported by Cienciactiva from Concytec, Peru. Project grant J004-2016 and contract 112-2017.

VI. References

[1] C. L. Asplund, J. J. Todd, A. P. Snyder, and R. Marois, "A central role for the lateral prefrontal cortex in goal-directed and stimulus-driven attention," *Nature Neuroscience,* vol. 13, no. 4, pp. 507-514, 2010.

[2] P. Giacometti, K. L. Perdue, and S. G. Diamond, "Algorithm to find high density EEG scalp coordinates and analysis of their correspondence to structural and functional regions of the brain," *Journal of Neuroscience Methods,* vol. 229, pp. 84-96, 2014.

[3] A. Sepúlveda, M. Linné, J. Llamas Alonso, M. A. Guevara, and M. Hernández González, "Increased prefrontal-parietal eeg gamma band correlation during motor imagery in expert video game players," *Actualidades en Psicología Vol. 28 Núm. 117 2014,* 2014.

[4] M. M. Smith, K. E. Weaver, T. J. Grabowski, R. P. Rao, and F. Darvas, "Non-invasive detection of high gamma band activity during motor imagery," *Frontiers in human neuroscience,* vol. 8, p. 817, 2014.

[5] X. Chia, J. B. Hagedorna, D. Schoonovera, and M. D'Zmuraa, "EEG-Based Discrimination of Imagined Speech Phonemes," *International Journal of Bioelectromagnetism,* vol. 13, no. 4, pp. 201-206, 2011.

[6] M. Fukuda, R. Rothermel, C. Juhász, M. Nishida, S. Sood, and E. Asano, "Cortical gamma-oscillations modulated by listening and overt repetition of phonemes," *Neuroimage,* vol. 49, no. 3, pp. 2735–2745, 2010.

[7] V. L.Towle *et al.*, "ECoG gamma activity during a language task: differentiating expressive and receptive speech areas," *Brain,* vol. 131, pp. 2013-2027, 2008.

[8] X. Pei, E. C. Leuthardt, C. M. Gaona, P. Brunner, J. R. Wolpaw, and G. Schalk, "Spatiotemporal dynamics of electrocorticographic high gamma activity during overt and covert word repetition," *NeuroImage,* vol. 54, pp. 2960-2972, 2011.

[9] P. Indefrey and W. J. M. Levelt, "The spatial and temporal signatures of word production components," *Cognition,* vol. 92, pp. 101-144, 2004.

[10] E. C. Leuthardt *et al.*, "Temporal evolution of gamma activity in human cortex during an overt and covert word repetition task," *Front Hum Neurosci,* vol. 6, p. 99, 2012.

[11] E. Kraft, B. Gulyas, and E. Poppel, *Neural Correlates of Thinking*. Berlin: Springer-Verlag, 2009, p. 285.

[12] M. Korostenskaja, C. Kapeller, K. H. Lee, C. Guger, J. Baumgartner, and E. M. Castillo, "Characterization of cortical motor function and imagery-related cortical activity: Potential application for prehabilitation," *arXiv preprint arXiv:1708.00525,* 2017.

[13] A. Jahangiri and F. Sepulveda, "The contribution of different frequency bands in class separability of covert speech tasks for BCIs," in *Engineering in Medicine and Biology Society (EMBC), 2017 39th Annual International Conference of the IEEE*, 2017, pp. 2093-2096: IEEE.

[14] G. Ruffini *et al.*, "ENOBIO dry electrophysiology electrode; first human trial plus wireless electrode system," in *Engineering in Medicine and Biology Society, 2007. EMBS 2007. 29th Annual International Conference of the IEEE*, 2007, pp. 6689-6693: IEEE.

[15] A. Delorme and S. Makeig, "EEGLAB: an open source toolbox for analysis of single-trial EEG dynamics including independent component analysis," *J Neurosci Methods,* vol. 134, no. 1, pp. 9-21, Mar 15 2004.

[16] G. Gómez-Herrero, *Automatic artifact removal (AAR) toolbox v1. 3 for MATLAB.* 2007.

[17] G. Gomez-Herrero *et al.*, "Automatic Removal of Ocular Artifacts in the EEG without an EOG Reference Channel," in *Signal Processing Symposium NORSIG*, 2006, pp. 130-133.

[18] X. Chen, C. He, and H. Peng, "Removal of Muscle Artifacts from Single-Channel EEG Based on Ensemble Empirical Mode Decomposition and Multiset Canonical Correlation Analysis," *Journal of Applied Mathematics,* p. 10, 2014.

[19] B. W. McMenamin, A. J. Shackman, L. L. Greischar, and R. J. Davidson, "Electromyogenic artifacts and electroencephalographic inferences revisited," *NeuroImage,* vol. 54, pp. 4-9, 2011.

[20] B. W. McMenamina *et al.*, "Validation of ICA-Based Myogenic Artifact Correction for Scalp and Source-Localized EEG," *Neuroimage,* vol. 49, no. 3, pp. 2416-2432, 2010.

[21] Q. Shie and C. Dapang, "Optimal biorthogonal analysis window function for discrete Gabor transform," *IEEE Transactions on Signal Processing,* vol. 42, no. 3, pp. 694-697, 1994.

[22] S. Qian and D. Chen, "Discrete Gabor transform," *IEEE Transactions on Signal Processing,* vol. 41, no. 7, pp. 2429-2438, 1993.

[23] A. R. Webb, *Statistical Pattern Recognition.* John Wiley and Sons Ltd., 2002.

[24] D. L. Davies and D. W. Bouldin, "A cluster separation measure," *IEEE Trans Pattern Anal Mach Intell,* vol. 1, no. 2, pp. 224-7, Feb 1979.

[25] P. Indefrey, "The spatial and temporal signatures of word production components: a critical update," *Frontiers in Psychology,* vol. 2, no. 255, pp. 255-271, 2011.

[26] J. S. Brumberg, E. J. Wright, D. S. Andreasen, F. H. Guenther, and P. R. Kennedy, "Classification of intended phoneme production from chronic intracortical microelectrode recordings in speech-motor cortex," *Front Neurosci,* vol. 5, p. 65, 2011.

[27] A. Llorens, A. Trebuchon, C. Liegeois-Chauvel, and F. X. Alario, "Intra-cranial recordings of brain activity during language production," *Front Psychol,* vol. 2, p. 375, 2011.

[28] J. D. Greenlee *et al.*, "Human Auditory Cortical Activation during Self- Vocalization," *PLoS One,* vol. 6, no. 3, pp. 1-15, 2011.

[29] A. Basirat, M. Sato, J. L. Schwartz, P. Kahane, and J. P. Lachaux, "Parieto-frontal gamma band activity during the perceptual emergence of speech forms," *Neuroimage,* Research Support, Non-U.S. Gov't vol. 42, no. 1, pp. 404-13, Aug 1 2008.
